# Tratando Números ou Pacientes? Repensando a Hipertensão Supina em Adultos Hospitalizados

**DOI:** 10.36660/abc.20250324

**Published:** 2025-11-26

**Authors:** Cassiano Teixeira

**Affiliations:** 1 Complexo Hospitalar da Santa Casa de Porto Alegre Hospital Nora Teixeira ICU Porto Alegre RS Brasil ICU – Hospital Nora Teixeira – Complexo Hospitalar da Santa Casa de Porto Alegre, Porto Alegre, RS – Brasil; 2 Hospital de Clínicas de Porto Alegre ICU Porto Alegre RS Brasil ICU – Hospital de Clínicas de Porto Alegre, Porto Alegre, RS – Brasil; 3 Universidade Federal de Ciências da Saúde de Porto Alegre Faculdade de Medicina Porto Alegre RS Brasil Universidade Federal de Ciências da Saúde de Porto Alegre – Faculdade de Medicina, Porto Alegre, RS – Brasil

**Keywords:** Hipertensão, Pacientes Internados, Hipotensão Ortostática

## Abstract

O monitoramento da pressão arterial (PA) em ambiente hospitalar frequentemente leva à detecção de leituras elevadas isoladas, particularmente na posição supina durante verificações noturnas. No entanto, evidências crescentes sugerem que esses valores hipertensivos transitórios – frequentemente denominados "hipertensão supina" – podem não justificar intervenção farmacológica na ausência de lesão aguda em órgão-alvo. Este artigo de opinião argumenta contra o tratamento reflexivo da hipertensão supina em pacientes hospitalizados e destaca os riscos associados ao manejo agressivo. O monitoramento excessivo e a falta de interpretação contextual das flutuações da PA podem expor os pacientes a danos desnecessários.

São 2 da manhã e uma enfermeira registra uma PA de 178/96 mmHg em um paciente deitado tranquilamente na cama. Sem sintomas. Sem sinais de lesão em órgão-alvo. Mesmo assim, o dado é sinalizado, um médico é chamado e, em poucas horas, um anti-hipertensivo é prescrito. Parece familiar?

Em hospitais modernos, onde o monitoramento contínuo é rotineiro e os alertas são automatizados, esse cenário é cada vez mais comum. No entanto, faz sentido tratar uma leitura isolada da PA em decúbito dorsal em um paciente sem queixas cardiovasculares e sem urgência hipertensiva? Evidências sugerem não apenas que isso pode ser desnecessário, mas também prejudicial.^1–3^ Pacientes hospitalizados frequentemente são submetidos à medição da pressão arterial (PA) em horários e posições que não refletem suas normas fisiológicas. Uma revisão sistemática histórica descobriu que 50% a 70% dos pacientes internados apresentam pelo menos uma medição elevada da PA (> 140/90 mmHg) durante a hospitalização.^4^ Essas leituras geralmente ocorrem durante as horas noturnas, na posição supina, e são influenciadas por estresse, dor, ansiedade ou até mesmo retenção urinária. De fato, estudos mostram que a pressão sistólica na posição supina pode ser até 8 mmHg maior do que na posição sentada.^5–7^ Essa disparidade alimentou uma epidemia silenciosa: o tratamento excessivo da hipertensão transitória em pacientes internados. A dependência de limiares automatizados em vez do julgamento clínico pode levar os médicos a agirem de forma reflexiva. No entanto, nem todos os números precisam ser perseguidos. Tratar elevações da PA em hospitais como se fossem diagnósticos ambulatoriais corre o risco de medicalizar as flutuações fisiológicas normais.

Vários estudos demonstraram que a intensificação da terapia anti-hipertensiva em pacientes sem evidências de danos em órgãos-alvo não melhora os resultados a longo prazo e pode, de fato, levar a danos.^1–3^ Relataram que pacientes internados cujos regimes foram intensificados durante a hospitalização não apresentaram melhor controle da PA um ano depois. Pior ainda, eles apresentaram maiores taxas de lesão renal aguda, síncope e transferências não planejadas para a UTI.^1,2^ A justificativa fisiológica é simples. Muitos pacientes hospitalizados — especialmente idosos e frágeis — apresentam respostas autonômicas prejudicadas. A redução abrupta da PA, particularmente à noite, pode inclinar a balança para hipoperfusão cerebral, quedas ou isquemia miocárdica. A hipercorreção farmacológica da hipertensão supina assintomática, neste contexto, não é apenas desnecessária — pode ser perigosa.^8,9^

Antes de iniciar o tratamento para hipertensão em decúbito dorsal, se deve descartar a hipotensão ortostática, uma condição comum, porém subdiagnosticada, em pacientes hospitalizados. A hipotensão ortostática é definida como uma queda sustentada da PA sistólica ≥ 20 mmHg ou da PA diastólica ≥ 10 mmHg em até três minutos após a posição ortostática.^10–12^ O tratamento da PA supina sem avaliar os valores em posição ortostática pode mascarar essa vulnerabilidade subjacente. Em pacientes com hipotensão ortostática neurogênica, o tratamento agressivo da hipertensão em decúbito dorsal pode exacerbar a hipotensão diurna e provocar síncope ou quedas.^13,14^ A elevação da cabeceira da cama, refeições pequenas e frequentes e horários de medicação noturnos costumam ser mais apropriados do que anti-hipertensivos padrão nesses casos.^15^

A obsessão por números em detrimento do contexto está no cerne desta questão. As leituras da PA devem ser interpretadas no contexto clínico em que foram obtidas. Foram aferidas durante dor? Durante ansiedade? À noite, em um quarto escuro com atividade mínima? O paciente estava com a bexiga cheia? Todos esses fatores podem elevar temporariamente a PA. Além disso, diferentemente do tratamento ambulatorial, em que a elevação consistente em múltiplas leituras é necessária para definir hipertensão, o diagnóstico em regime de internação frequentemente depende de um ou dois valores fora do contexto. Essa prática contradiz as recomendações das diretrizes atuais, que enfatizam a interpretação e o acompanhamento cautelosos em vez do tratamento impulsivo.^5–8,12^

Existem, é claro, situações em que a PA elevada em pacientes hospitalizados deve ser abordada. A presença de emergências hipertensivas – como acidente vascular cerebral agudo, infarto do miocárdio ou dissecção aórtica – justifica tratamento farmacológico imediato.^2^ Da mesma forma, pacientes com hipertensão sabidamente mal controlada e PA persistentemente elevada durante a admissão podem se beneficiar de ajustes em seu regime crônico. No entanto, na ausência de sintomas ou danos em órgãos-alvo, a hipertensão isolada em posição supina em pacientes internados por condições não cardiovasculares raramente justifica agentes anti-hipertensivos intravenosos ou mesmo escalonamento da dose oral.^13,15^ A estratégia de tratamento apropriada para a maioria dos casos de hipertensão em posição supina em pacientes internados é reconhecer a elevação, documentar a ausência de danos em órgãos-alvo e planejar o acompanhamento ambulatorial. Se a hipertensão for persistente em diferentes momentos e posições corporais, o monitoramento ambulatorial ou domiciliar da PA após a alta pode orientar as decisões terapêuticas a longo prazo. Esforços para intensificar a terapia em ambiente hospitalar frequentemente levam à alta dos pacientes com regimes desnecessariamente complexos. Esses regimes são frequentemente modificados ou descontinuados em poucas semanas, destacando a ineficácia e a instabilidade de tais decisões quando tomadas durante doenças agudas.^11,14^

A hipertensão em decúbito dorsal durante a hospitalização não deve desencadear um reflexo automático de prescrição. Em vez disso, os médicos devem recuar, considerar a postura, o contexto e a fisiologia subjacente e se perguntar: "O que estou realmente tratando?". O controle agressivo da PA em pacientes internados, particularmente em pacientes assintomáticos sem lesão em órgão-alvo, pode criar mais problemas do que soluções (Tabela 1). Devemos parar de deixar que as máquinas ditem nossas decisões médicas e começar a resgatar nosso julgamento clínico. Afinal, nem todo número exige uma intervenção – e, às vezes, o melhor tratamento é simplesmente esperar.

**Tabela 1:** Resumo dos principais estudos sobre o manejo da pressão arterial em pacientes internados

Estudo (Autor, Ano)	Jornal	Desenho do Estudo	Descoberta principal	Tamanho do efeito / valor p
Jordan e Biaggioni, 2002^15^	J Clin Hipertens. 2002;4(5):349–353	Revisão narrativa	A hipertensão supina pode piorar a OH; abordagem conservadora é preferida	-
Axon et al., 2011^4^	J Hosp Med. 2011;6(7):417–422	Revisão sistemática	Alta prevalência de hipertensão transitória em hospital (50–70%)	-
Pikilidou et al., 2010^16^	Hypertens Res. 2010; 33(10):995–999	Observacional	A hospitalização reduziu a pressão arterial sistólica diurna em comparação com as medidas ambulatoriais.	PAS média ↓ 5 mmHg, p < 0,05
Rastogi et al., 2021^1^	JAMA Estagiário Med. 2021;181(3):373–381	Coorte observacional	Nenhum benefício no controle a longo prazo; ↑ Transferências de IRA e UTI	OU 1,34 para dano, p < 0,001
Anderson et al., 2023^17^	JAMA Intern Med. 2023;183(7):715-23	Coorte retrospectiva	Mais eventos adversos em idosos tratados intensivamente	HR 1,52 para IRA, p < 0,05
Anderson et al., 2023^2^	JAMA Estagiário Med. 2023;183(7):715–723	Coorte retrospectiva	Redução intensiva da PA em pacientes internados associada à mortalidade e eventos cardiovasculares	OU 1,26 para dano, p = 0,01
Malhotra et al., 2023^3^	Hipertensão. 2023;80(6):1256–1263	Coorte prospectiva	Hipertensão supina associada a ↑ eventos cardiovasculares	HR 2,09, IC 95%: 1,18–3,68

IRA: lesão renal aguda; HR: razão de risco; HTA: hipertensão; UTI: unidade de terapia intensiva; OH: hipotensão ortostática; OR: razão de chances; PAS: pressão arterial sistólica.

## Data Availability

Os conteúdos subjacentes ao texto da pesquisa estão contidos no manuscrito.
