# Supplementary material for: Tratando Números ou Pacientes? Repensando a Hipertensão Supina em Adultos Hospitalizados
Source: Arq Bras Cardiol. 2025 Nov 26;122(9):e20250324. [Article in Portuguese] doi: 10.36660/abc.20250324 (PMC12671725; doi:10.36660/abc.20250324)
Supplement: Translation

# Translation: Treating Numbers or Patients? Rethinking Supine Hypertension in Hospitalized Adults

**DOI:** 10.36660/abc.20250324i

**Authors:** Cassiano Teixeira

**Affiliations:** 1 Porto Alegre RS Brazil ICU – Hospital Nora Teixeira – Complexo Hospitalar da Santa Casa de Porto Alegre, Porto Alegre, RS – Brazil; 2 Porto Alegre RS Brazil ICU – Hospital de Clínicas de Porto Alegre, Porto Alegre, RS – Brazil; 3 Porto Alegre RS Brazil Universidade Federal de Ciências da Saúde de Porto Alegre – Faculdade de Medicina, Porto Alegre, RS – Brazil

**Keywords:** Hipertensão, Pacientes Internados, Hipotensão Ortostática

## Abstract

In-hospital blood pressure (BP) monitoring often leads to the detection of isolated elevated readings, particularly in the supine position during nighttime checks. However, growing evidence suggests that these transient hypertensive values - frequently labeled as "supine hypertension" - may not warrant pharmacologic intervention in the absence of acute end-organ damage. This opinion piece argues against the reflexive treatment of supine hypertension in hospitalized patients and highlights the risks associated with aggressive management. Excessive monitoring and the lack of contextual interpretation of BP fluctuations may expose patients to unnecessary harm.

It's 2 a.m., and a nurse notes a BP of 178/96 mmHg in a patient lying quietly in bed. No symptoms. No signs of end-organ damage. Yet the chart gets flagged, a physician is paged, and within hours, an antihypertensive is prescribed. Sound familiar?

In modern hospitals, where continuous monitoring is routine and alerts are automated, this scenario is increasingly common. Nevertheless, does it make sense to treat an isolated supine blood pressure (BP) reading in a patient with no cardiovascular complaint and no hypertensive urgency? Evidence suggests not only that it may be unnecessary, but that it could be harmful.^1–3^ Hospitalized patients frequently undergo BP measurement at times and in positions that do not reflect their physiological norms. A landmark systematic review found that 50% to 70% of inpatients experience at least one elevated BP measurement (>140/90 mmHg) during hospitalization.^4^ These readings often occur during nocturnal hours, in the supine position, and are influenced by stress, pain, anxiety, or even urinary retention. In fact, studies show that systolic pressure in the supine position can be up to 8 mmHg higher than in the seated position.^5–7^ This disparity has fueled a silent epidemic: the overtreatment of transient inpatient hypertension. The reliance on automated thresholds rather than clinical judgment can push physicians to act reflexively. However, not every number needs to be chased. Treating hospital-based BP elevations as though they were outpatient diagnoses risks medicalizing normal physiological fluctuations.

Several studies have shown that intensifying antihypertensive therapy in patients without evidence of end-organ damage does not improve long-term outcomes and may, in fact, lead to harm.^1–3^ Reported that inpatients whose regimens were intensified during hospitalization did not exhibit better BP control one year later. Worse, they had higher rates of acute kidney injury, syncope, and unplanned ICU transfers.^1,2^ The physiological rationale is simple. Many hospitalized patients – especially the elderly and frail - have impaired autonomic responses. Abrupt BP reduction, particularly at night, may tip the balance toward cerebral hypoperfusion, falls, or myocardial ischemia. Pharmacologic overcorrection of asymptomatic supine hypertension, in this context, is not just unnecessary - it may be dangerous.^8,9^

Before initiating therapy for supine hypertension, one must rule out orthostatic hypotension - a common yet underdiagnosed condition in hospitalized patients. Orthostatic hypotension is defined as a sustained drop in systolic BP of ≥20 mmHg or diastolic BP of ≥10 mmHg within three minutes of standing.^10–12^ Treating supine BP without assessing standing values may mask this underlying vulnerability. In patients with neurogenic orthostatic hypotension, aggressive treatment of supine hypertension may exacerbate daytime hypotension and provoke syncope or falls.^13,14^ Head-of-bed elevation, small frequent meals, and nighttime medication timing are often more appropriate than standard antihypertensives in these cases.^15^

The obsession with numbers over context is at the core of this issue. BP readings should be interpreted in the clinical setting in which they were obtained. Were they taken during pain? While anxious? At night in a dark room with minimal activity? Did the patient have a full bladder? All these factors can temporarily drive BP up. Moreover, unlike outpatient management, where consistent elevation over multiple readings is necessary to define hypertension, inpatient diagnosis often hinges on one or two out-of-context values. This practice contradicts current guideline recommendations, which emphasize cautious interpretation and follow-up rather than knee-jerk treatment.^5–8,12^

There are, of course, situations where elevated BP in hospitalized patients must be addressed. The presence of hypertensive emergencies - such as acute stroke, myocardial infarction, or aortic dissection - warrants prompt pharmacologic treatment.^2^ Similarly, patients with known poorly controlled hypertension and persistently elevated BP during admission may benefit from adjustments to their chronic regimen. However, in the absence of symptoms or end-organ damage, isolated supine hypertension in patients admitted for non-cardiovascular conditions rarely justifies intravenous antihypertensive agents or even oral dose escalation.^13,15^ The appropriate management strategy for most cases of inpatient supine hypertension is to acknowledge the elevation, document the absence of end-organ damage, and plan outpatient follow-up. If hypertension is persistent across different times and body positions, ambulatory or home BP monitoring after discharge can guide long-term therapy decisions. Efforts to ramp up therapy in the inpatient setting often lead to patients being discharged on unnecessarily complex regimens. These regimens are frequently modified or discontinued within weeks, highlighting the inefficacy and instability of such decisions when made during acute illness.^11,14^

Supine hypertension during hospitalization should not trigger an automatic prescribing reflex. Instead, clinicians must step back, consider posture, context, and underlying physiology, and ask themselves: "What am I really treating?" Aggressive inpatient BP control, particularly in asymptomatic patients without end-organ injury, may create more problems than it solves (Table 1). We must stop letting machines dictate our medical decisions and start reclaiming our clinical judgment. After all, not every number demands an intervention - and sometimes, the best treatment is simply to wait.

**Table 1:** Summary of Key Studies on Inpatient Blood Pressure Management

Study (Author, Year)	Journal	Study Design	Main Finding	Effect Size / p-value
Jordan e Biaggioni, 2002^15^	J Clin Hypertens. 2002;4(5):349–353	Narrative review	Supine HTN may worsen OH; conservative approach preferred	-
Axon et al., 2011^4^	J Hosp Med. 2011;6(7):417–422	Systematic review	High prevalence of transient hypertension in hospital (50–70%)	-
Pikilidou et al., 2010^16^	Hypertens Res. 2010; 33(10):995–999	Observational	Hospitalization reduced daytime systolic blood pressure compared with outpatient measurements	Mean SBP ↓ 5 mmHg, p < 0.05
Rastogi et al., 2021^1^	JAMA Intern Med. 2021;181(3):373–381	Observational cohort	No benefit in long-term control; ↑ AKI and ICU transfers	OR 1.34 for harm, p < 0.001
Anderson et al., 2023^17^	JAMA Intern Med. 2023;183(7):715-23	Retrospective cohort	More adverse events in intensively treated older adults	HR 1.52 for AKI, p < 0.05
Anderson et al., 2023^2^	JAMA Intern Med. 2023;183(7):715–723	Retrospective cohort	Intensive inpatient BP lowering linked to ↑ mortality and cardiovascular events	OR 1.26 for harm, p = 0.01
Malhotra et al., 2023^3^	Hypertension. 2023;80(6):1256–1263	Prospective cohort	Supine HTN associated with ↑ cardiovascular events	HR 2.09, 95% CI: 1.18–3.68

AKI: acute kidney injury; HR: hazard ratio; HTN: hypertension; ICU: intensive care unit; OH: orthostatic hypotension; OR: odds ratio; SBP: systolic blood pressure.

## Data Availability

The underlying content of the research text is contained within the manuscript
